# Cocaine in Surgery

**Published:** 1893-04

**Authors:** Jules Auber

**Affiliations:** Paris


### *SELECTED ARTICLE.*

### COCAINE IN SURGERY.

By JULES AUBER,
PARIS.

Dr. Jules Auber has published a pamphlet on the use of cocaine in surgery, of which the following is an abstract:

Cocaine was first extracted from the leaves of the Erythroxylon *Coca* by Nieman in 1859. In 1862 Dr. Schroeff pointed out for the first time its anæsthetic and analgesic properties. In 1868 Morenoy Mais pointed out that cocaine in large doses causes in animals the diminution, afterwards the loss of feeling, without movement being completely abolished, the pupil remaining dilated in every case. Karl Koeller, in 1884, at the Ophthalmological Congress at Heidelberg, first pointed out the therapeutic uses of cocaine on the eye.

Cocaine is a toxic substance, like most other alkaloids, and is accordingly included in the class of powerfully active remedies, which for this very reason demand prudence in their administration. Whilst much neglected in most places, Dr. Albers, at a Medical Congress at Heidelberg, points out that for four years he has always employed solutions of 5 per cent., and always with the greatest success in a considerable number of severe operations. His conclusions are:

(1) Solutions of cocaine (5 per cent.) form the best local anæsthetic for large skin incisions and the removal of large tumors.

(2) Solutions stronger than 5 per cent. are not useful, and should be rejected.

(3) Cocaine injections of 5 per cent., made in the manner pointed out below, ought to be preferred to chloroform by reason of their harmlessness and their rapidity of action.

(4) Chloroform should have the preference in children and delicate persons.

Other surgeons, on the other hand, have exaggerated the dangers of cocaine, and Dr. Auber has, with the view of coming to a correct conclusion, analyzed all the published cases of fatality from cocaine.

Sixteen cases are summarized, and as two were reported twice, and one was not due to cocaine, thirteen remain. Three other cases are excluded, where the cocaine was administered, not by hypodermic injection, but by the mouth. In one other case the dose injected was not known. In four the dose varied from 22 to 150 centigrammes, which is far too large, and a fatal result is almost a natural consequence. Of the three last, in two there was merely a coincidence, and the third was proved to have nothing to do with the cocaine.

As regards the non-fatal cases of poisoning, the symptoms generally were: Extreme pallor of face, quickening of the heart's action, respiration frequent and superficial, præcordial anxiety, dilatation of the pupils, feeling of approaching dissolution, complete unconsciousness, collapse and coma.

A striking fact is the variability of the dose causing poisonous symptoms ; this depends on several conditions, *e. g.* :

(1) The seat of injection. Rectum, face and head appear more dangerous than the extremities.

(2) The strength of the solution is of importance, and should be strictly noted.

(3) The condition of the organs with which the cocaine comes in contact. The wall of a recent hydrocele absorbs much better than one where the tunica vaginalis is much thickened. The mucous membrane of a healthy bladder does not absorb, but is very different when deprived of its epithelium.

(4) Emotion may stimulate the effects of cocaine. A case of M. Hugenschmidt is cited, where a patient who had been told often of the injection of cocaine, had injected without knowing it some distilled water. In a few seconds she complained of pains in the head, walked a few steps and fell in a faint into a chair; syncope lasted for half an hour.

As regards the objection that cocaine is not trustworthy, and is useless for prolonged operations, Dr. Auber observes that it is necessary to obtain a pure drug, and secondly to know and follow exactly the rules laid down for the injection of the drug. Cocaine is also quite efficacious in cases of inflamed tissues, in lymphangitis or in whitlows. As regards the bones, the following operations are quoted: (1) Amputation of the forearm; (2) resection of the heads of the right and left metatarsal bones; (3) osteomyelitis of the tibia; (4) amputation of the great toe and its metatarsal bone; (5) traumatic osteitis of the right great toe (resection); (6) osteitis of the index finger of the right hand (resection); (7) fracture of the patella (suture with silver wire); (8) caries of the ribs; (9) Dr. T. R. Varick reports in the *New York Medical Journal* (February 20) a case of amputation of the thigh by means of cocaine.

"In short the complaints about cocaine are illusory; its advantages, on the contrary, inestimable. With cocaine, a prick, and that is all, instead of the struggle and excitement with chloroform, and the many alarming accidents interfering with the operation—accidents terminating often in death, and followed by feebleness and vomiting, often so serious and prejudicial to the union of the part operated on. The patient is not made ill; far from that, he himself helps you, 'he places himself here and there, and obeys your orders.' Also in the country, where help cannot be obtained, how much easier to operate by cocaine than chloroform? Lastly, cocaine is indispensable in small operations, such as hemorrhoids and fissure of the anus."

*Method of Application.*—The surgeon here, as in all other cases, should be perfectly aseptic; hands, forearms, etc. In addition, he must show no fear, or he might alarm the patient and by suggestion cause symptoms, as in the case quoted where distilled water was injected.

As regards the patient—

(1) To take notice of the constitution, to be more careful with those who are anæmic or hysterical, in aortic diseases (stenosis and regurgitation) on account of their tendency to produce cerebral anæmia, and also in true angina pectoris caused by ischæmia of the myocardium.

(2) The thorax must be free from everything which could possibly interfere with respiratory movements.

(3) The horizontal position is absolutely necessary during the operation and afterwards. Cocaine acts on the encephalic vaso-motor nerves, and tends to produce cerebral anæmia.

(4) It is much better to make the injections during the period of digestion than fasting.

(5) The patient must drink a small glass of brandy half an hour before the operation, and a second during the operation, if he seems pale.

(6) It is well to make the syringe antiseptic before use.

(7) For the preparation of the solution of cocaine, we use pure water recently boiled, and consequently sterilized.

(8) As to the dose, it should not exceed 6, 8 or 10 centigrammes, which is sufficient for the most extensive operation; larger doses are certainly useless. But what is of the utmost importance is the strength for the solution; a 2 per cent. solution in small operations, those which require in all 2 to 4 centigrammes of cocaine; a 1 per cent. solution for those which require 6, 8 or 10 centigrammes. It appears to be established that the dangers of cocaine depend much more on the strength of the solution than on the quantity of cocaine injected.

*Rules for Injection.*—Along the line of the intended incision, with the needle of the hypodermic syringe, make a prick in the skin; but in order that it may not go through take care to give the needle a direction almost parallel to the skin. As soon as the needle is in the thickness of the dermis, push the piston of the syringe, and press out a few drops of the liquid; thence, if the needle advances slowly, its passage is not perceived by the patient, for the cocaine coming out at the point anæsthetizes the tissues where the point penetrates. Then push the needle slowly forwards, with the forefinger on the piston, and the needle goes along the thickness of the skin, which always presents a certain resistance; if it go suddenly, the needle has penetrated the cellular tissue, and in this case must be brought parallel again until the new resistance proves that the needle has re-entered into the dermis. Two signs point out the proper course of the needle: the skin puffs up slightly following the line of injection, then it becomes pale and gets a livid tint.

If the incision is extensive either use a long needle or reintroduce the needle again and again where the former injection ceases.

The injection is now finished; if the solution used is a 2 per cent. one may take the knife immediately; if a 1 per cent. the analgesic effect is slower in being produced, and it necessary to wait five or six minutes.

When operating on the limbs the use of Esmarch's bandage may assist in preventing accidents.

In case of symptoms of poisoning, which should be exceedingly rare, if the before-mentioned precautions are carried out, we must rely on inhalation of nitrite of amyl and ammonia, and the administration of brandy and coffee. It has also been proposed to employ atropine and chloral.

In addition one must not forget to watch the breathing, and in case of need to perform artificial respiration.

Full directions are given for the procedure in various operations, and then a table of ninety operations of all kinds performed under cocaine, including hemorrhoids, fistula, radical cure of hernia and hydrocele, varicocele, phymosis, vaginal hysterectomy, removal of hydatid cysts and tumors, resection of bones and amputation of fingers and toes. The conclusions of the author are summarized as follows:

(1) The dose of cocaine injected ought to be proportional to the extent of surface to be operated upon; it will never in any case exceed 8 to 10 centigrammes, and this only in cases of large surfaces.

(2) The strength of the solution will be 2 per cent. for small operations, and 1 per cent. for large operations.

(3) The cocaine must be injected into the skin, and not under the dermis of the mucous membrane or skin; *i. e.*, it must be intradermic.

We must push on the needle at the same time we push down the piston, in order to avoid the risk of injecting the whole of the fluid into a vein.

(4) The patient must take care to keep the horizontal position both during and after the operation, and the room must not be crowded.—*Medical Chronicle.*

